# Carboplatin, paclitaxel, and pembrolizumab followed by pembrolizumab maintenance for primary treatment of incompletely resected epithelial ovarian cancer

**DOI:** 10.3389/fonc.2024.1291090

**Published:** 2024-02-12

**Authors:** Denise Uyar, Chad M. Michener, Erin Bishop, Elizabeth Hopp, Pippa Simpson, Liyun Zhang, Janet S. Rader, Peter G. Rose, Haider S. Mahdi, Robert Debernardo, Qiana Christian, William Bradley

**Affiliations:** ^1^ Department of Obstetrics and Gynecology, Medical College of Wisconsin, Milwaukee, WI, United States; ^2^ Obstetrics and Gynecology Institute, Cleveland Clinic, Cleveland, OH, United States

**Keywords:** ovarian cancer, chemotherapy, immunotherapy, incomplete resection, gynecologic oncologic surgery

## Abstract

**Objective:**

Incompletely resected epithelial ovarian cancer represents a poor prognostic subset of patients. Novel treatment strategies are needed to improve outcomes for this population. We evaluated a treatment strategy combining platinum-based chemotherapy with pembrolizumab followed by pembrolizumab maintenance therapy in the first-line treatment after incomplete resection of epithelial ovarian cancer patients.

**Methods:**

This was a single-arm, non-randomized pilot study of carboplatin, taxane, and immune checkpoint inhibitor, pembrolizumab, followed by 12 months of maintenance pembrolizumab in patients with incompletely resected epithelial ovarian cancer (EOC).

**Results:**

A total of 29 patients were enrolled and evaluated for efficacy and safety. The best response to therapy was complete response in 16 (55%) patients, partial response in 9 (31%) patients, and 3 (10%) patients with progression of disease. The median progression-free survival (PFS) was 13.2 months. Grade 3 and 4 toxicities occurred in 20% of patients. In all, 7 patients discontinued therapy due to adverse events. Quality-of-life scores remained high during therapy. Response to therapy did not correlate with PD-L1 tumor expression.

**Conclusions:**

Combination platinum–taxane therapy with pembrolizumab did not increase median progression-free survival in this cohort of patients.

**Key message:**

EOC is an immunogenic disease, but immune checkpoint inhibitor therapy has yet to impact outcomes. The current study utilized pembrolizumab in combination with standard chemotherapy followed by a maintenance treatment strategy in incompletely resected EOC. Progression-free survival was not extended in this poor prognostic group with combined chemotherapy and immunotherapy.

**Clinical trial registration:**

https://clinicaltrials.gov/, identifier NCT 027766582.

## Introduction

The cornerstones of treatment for epithelial ovarian cancer (EOC) remain cytoreductive surgery and platinum–taxane-based chemotherapy. In selected patients, three to four cycles of neoadjuvant platinum-based chemotherapy followed by interval cytoreductive surgery and then additional chemotherapy is a well-established alternative. The determination of which patients would benefit most from the neoadjuvant approach is still being debated (1). Many patients will nevertheless undergo primary cytoreductive surgery followed by platinum-based chemotherapy. Optimal cytoreduction is defined as < 1 cm residual disease after surgical resection and is associated with superior survival outcomes (2). Complete cytoreductive surgery or microscopic-only *residual disease* (R0) correlates with significant median progression-free and overall survival in both the primary and interval setting and remains the ideal (3–6). Although the definitions have evolved, patients with *any* macroscopic residual disease may be considered incompletely resected and represent a poor prognostic group. Even in the platinum era, patients whose surgical efforts result in incomplete cytoreduction have some of the poorest survival outcomes, with a median progression-free survival of 33 months in patients with < 1mm residual disease compared to 16.8 months and 14 months in patients with 1 mm-10 mm and > 10 mm residual disease, respectively (3).

Incomplete resection of EOC may be attributed to many diverse factors, but in terms of tumor microenvironment, it could represent the presence of greater inflammation and even greater altered tumor microenvironment.

Epithelial ovarian cancer is thought to be a very heterogeneous disease. Distinct separation has been made of type I and type II EOC, which develop along two separate carcinogenic pathways. Type I is a more indolent procession of events, with identifiable precursor lesions, typically characterized by mutations of *KRAS*, *BRAF*, and *ERBB2*. Type II is the more common type of EOC, categorized as highly aggressive (i.e., high-grade serous EOC),with *p53* and *BRCA* mutations playing a significant role in their development (7). BRCA1 and BRCA2 germline mutations are found in 6-15% of women with HGSOC (8) and have been found to confer greater platinum sensitivity.

Type II high-grade serous ovarian cancer (HGSOC) is thought to be an immunogenic disease (9, 10). Increased infiltration of T cells in tumor islets correlates with significantly longer survival (9, 10). Approximately half of the patients with ovarian cancer demonstrate T-cell infiltration in the tumor microenvironment (TME). It has been shown that T cells are subjected to various mechanisms of suppression such as FoxP3 regulatory T cells and expression of programmed cell death inhibition, which diminishes their anti-tumor responses (11, 12). Cytotoxic therapy and specifically platinum-based therapy have been shown to stimulate the immune system. Programmed cell death 1 (PD-1) and its ligand (PD-L1) are expressed in 28-40% of patients with ovarian cancer (13, 14), and thus, anti-PD-1 therapy could be considered a rational strategy for the targeted treatment of ovarian cancers. While the initial results of monotherapy with programmed death-ligand 1 (PD-1) immune checkpoint inhibitors in ovarian cancer were encouraging (13, 15), minimal durable success to date has been noted (16). Many have suggested that monotherapy with checkpoint blockade is insufficient and that combination therapy is necessary to elicit a maximum antitumor response (17, 18). Early data confirmed the safety of combining pembrolizumab and platinum/paclitaxel therapy in non-small cell lung cancer, with efficacy noted regardless of the PD-L1 tumor expression (19). This opened the door to the exploration of additional combination strategies.

The primary aim of our study was to examine the progression-free survival of platinum-based chemotherapy with the anti-PDL1 inhibitor, pembrolizumab, followed by 12 months of pembrolizumab maintenance therapy in patients with incompletely resected EOC, given that this population represents a very poor prognostic cohort and is in great need of alternative strategies of treatment. The secondary aims of the study were to collect adverse events and immune-related adverse events related to the treatment and to assess patient QOL scores while on treatment.

## Materials and methods

### Study design and participants

This was an investigator-initiated, single-arm, non-randomized pilot study of carboplatin and taxane therapy combined with pembrolizumab therapy in patients with incompletely resected epithelial ovarian cancer after primary cytoreduction, followed by 12 months of pembrolizumab maintenance therapy. The study was a multisite investigation conducted at the Medical College of Wisconsin and the Cleveland Clinic. Eligible patients had newly diagnosed untreated International Federation of Gynecology and Obstetrics (FIGO) stage III or IV epithelial ovarian, fallopian tube, or primary peritoneal cancer (EOC) and had undergone primary cytoreductive surgery resulting in gross (macroscopic) or palpable (operative report documentation) residual disease. Additional eligibility included: age ≥ 18, Eastern Cooperative Oncology Group (ECOG) performance status ≤ 2, adequate hematologic, renal, and hepatic function; and availability of formalin-fixed, paraffin-embedded tumor specimen for evaluation of PD-L1 status. Patients with borderline epithelial ovarian tumors, non-epithelial tumors, or contraindications to pembrolizumab were not eligible. All patients provided written informed consent prior to study participation.

### Procedures

All patients received intravenous systemic therapy consisting of platinum-based therapy with carboplatin AUC 5-6 and a taxane consisting of paclitaxel 135 mg -175 mg/m2 every 21 days or paclitaxel 60-80 mg/m2 weekly. Physician choice determined paclitaxel every 21 days versus weekly paclitaxel treatment. Docetaxel (60- 75mg/m2) was utilized where a paclitaxel reaction or severe paclitaxel toxicity necessitated therapeutic exchange. The maximum number of platinum-based systemic therapy was eight cycles. Pembrolizumab was administered intravenously at a flat dose of 200 mg on day 1 of each cycle. All medications were administered every 21 days unless the patient was administered a weekly paclitaxel regimen or experienced toxicity requiring delay or discontinuation of therapy.

Physical examinations were performed every 3 weeks to assess treatment tolerance, response, and safety. Adverse events (AEs) were assessed at each cycle and graded according to the Common Terminology Criteria for Adverse Events (CTCAE) version 4.0.

All patients underwent baseline computed tomographic scans prior to initiation of therapy. The residual disease information was collected from surgical operative reports and computed tomographic (CT) scans; this is reported with demographic data in **Table 1**. Treatment responses were assessed with CA 125 at each cycle of therapy. CT scans were performed post-operatively prior to initiation of systemic therapy, at the completion of combination platinum, taxane, and pembrolizumab therapy, at the completion of maintenance pembrolizumab therapy. CT scans were also performed with increasing CA 125 or if clinically indicated per treating physician, and assessments were made using the response evaluation criteria in solid tumor (RECIST).

**Table 1:** Patient characteristics.

Demographics (total n = 29)	
Age, median (IQR)	64.0 (58.0, 70.0)
Race, n (%)	
White Black/African American	28 (96.6) 1 (3.4)
Primary cancer site, n (%)	
Ovarian Fallopian tube Peritoneal	26 (89.7) 2 (6.9) 1(3.4)
Stage	
IIIA IIIB IIIC IVA IVB	1 2 22 2 2
Histology, n (%)*	
carcinosarcoma clear cell endometrioid serous	1 (3.5) 3 (10.3) 2 (6.9) 23 (79.3)
Residual Disease	
1 mm < 10 mm > 10 mm - ≥ 20 mm > 20 mm	19 (65.5) 7 (24) 3 (10)
Current status, n (%)	
Progression (death) Progression (alive) Progression free (death) Progression free (alive)	8 (27.6) 11 (37.9) 2 (6.9) 8 (27.6)

*all high grade with exception of one patient with grade 1 endometrioid histology.

Quality-of-life assessment was conducted at baseline at the time of enrollment and at 3, 6, and 18 months from initiation of therapy using the Functional Assessment of Cancer Therapy – Ovarian (FACT-O) assessment, a validated 26-item summary score with a possible total of 112 points that captures the FACT-General (FACT-G) QOL dimensions of Physical Well-Being (7 items), Functional Well-Being (7 items), and an Ovarian Cancer Subscale (12 items) (20).

PD-L1 expression was determined from the tumor tissue sample obtained during primary cytoreductive surgery. PD-L1 staining was centrally performed by Qualtek Laboratories using the Merck 22C3 antibody for PD-L1 and reported through a modified percent score (MPS) ranging from 1 to 100. At the time of the study design and patient accrual, data on the clinical relevance of CPS were not yet available, and therefore CPS, was not used for correlative analysis. MPS scoring was employed across Merck’s Investigator Study Program and is comparable to CPS.

Germline genetic testing was performed on all participants.

This study was registered on ClinicalTrials.gov NCT 027766582.

This study received Institutional Review Board approval prior to initiation.

### Statistical analysis

Progression-free survival (PFS) is defined as the date of completion of primary therapy to the date of the first clinical, biochemical, or radiological evidence of progression or death due to any cause. PFS was censored at the last assessment of disease progression for living patients. The efficacy parameters of progression free survival were analyzed using Kaplan–Meier curves stratified by the group, in which the groups were compared using a log-rank sum test. Using Pass 12, the primary comparison is the PFS of the treated sample compared to historical controls. We planned to accrue patients over 3 years with a follow-up of at least 18 months. A sample size calculation was performed based on the work of Lakatos (21). The was a two-sided log-rank sum test, at an alpha of 0.05. Using the study by Katsumata, the median survival time was an estimated 18 months for the conventional carboplatin–paclitaxel chemotherapy regimen for those with >1cm remaining post-surgery. Realistically an increase of median PFS by 6 months would have indicated efficacy, but with a treatment sample of 30 and the control sample from Katsumata of 168, we would have at least 80% power to detect at least an increase for 1.5 years to ~3 years as an optimistic outcome (22).

A waterfall plot illustrates the maximum percent change in tumor measurement per RECIST from baseline. Continuous variables are summarized as median (interquartile range) and categorical variables and number (%). This included treatment-related adverse events assessed by the investigator as at least possibly related to treatment. FACTG scores were analyzed using a mixed-effects covariance pattern model to utilize all the data collected over time with consideration of the variance–covariance matrix of the repeated measures. This method allowed a general unstructured variance–covariance matrix and patients to have incomplete data across scheduled time points. All analyses were conducted using SAS 9.4 and SPSS 26.0.

## Results

A total of 29 patients were included in the final analysis, out of 33 patients who were screened for enrolment into the clinical trial. In all, 4 patients did not meet the eligibility criteria due to not meeting the laboratory criteria and performance status criteria and/or ultimately declining participation. The final population analyzed comprised 29 patients, all of whom had undergone tumor assessment at baseline, received at least one cycle of pembrolizumab in combination with first-line platinum-based chemotherapy, and had at least one post-dose tumor assessment.

Demographic data are provided in **Table 1**. The most common histologic subtype was high-grade serous. The volume of residual disease was 1-10 mm in 19 (66%) patients and > 10 mm residual disease in 10 (34%) patients. PD-L1 MPS scores ranged from 0 to 90%. MPS score > 1% was noted in 16 (55%) patients. Responses to therapy did not correlate with the MPS score and were observed regardless of PD L1 expression rates.

Toxicity associated with the regimen indicated that 20% of patients experienced grade 3 or 4 toxicity (**Table 2**). Most serious adverse events occurred during the combination therapy. In all, 7 patients (24%) discontinued therapy due to treatment-related adverse events, and 3 of these patients who discontinued therapy (all while receiving combination therapy) had toxicity that could possibly be attributable to immune-related adverse events: acute respiratory distress, pneumonitis, and congestive heart failure/pulmonary edema. Additionally, 4 patients discontinued therapy due to patient choice, renal toxicity, peripheral neuropathy, and metabolic toxicity respectively. The most frequently reported treatment-related toxicities at any grade were anemia (22 patients, 78.6%) and fatigue (23 patients, 79.3%). The study did not identify any new safety signals or unanticipated toxicities compared to the published data. No deaths were attributed to the investigational protocol during this study.

**Table 2:** Summary of adverse events.

Toxicities	Any grade, n (%)	Grades			
		1	2	3	4
**Lymphocyte**	8 (27.6)	6 (20.7)	4 (13.8)	3 (10.3)	1 (3.4)
**Anemia**	22 (78.6)	22 (75.9)	20 (67.0)	7 (24.1)	0
**Hypertension**	6 (20.7)	6 (20.7)	5 (17.2)	3 (10.3)	0
**Neutropenia**	17 (58.6)	11 (37.9)	10 (34.5)	11 (37.9)	3 (10.3)
**Acute Respiratory Distress Syndrome (ARDS)**	1 (3.4)	0	0	0	1 (3.4)
**Pneumonitis**	1 (3.4)	0	0	0	1 (3.4)
**Encephalopathy**	1 (3.4)	0	0	0	1 (3.4)
**Hyponatremia**	7 (24.1)	6 (20.7)	2 (6.9)	2 (6.9)	1 (3.4)
**Fatigue**	23 (79.3)	22 (78.6)	4 (13.8)	2 (6.9)	0
**Pulmonary edema**	1 (3.4)	0	0	0	1 (3.4)
**Hypothyroidism**	3 (10.3)	0	3 (10.3)	0	0
**Nausea**	2 (6.8)	0	0	2 (6.8)	0

**Figure 1:**
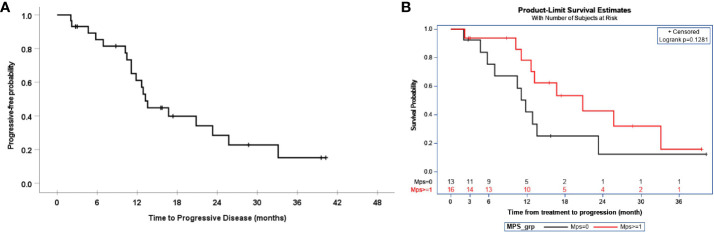
**(A)** Progression-free survival. **(B)** Progression-free survival PD-L1 MPS score.

The median and 95% CI of progression-free survival were 13.2 (11.8, 14.7) months, as demonstrated in **Figure 1A**. This time was comparable to the historical PFS of patients with 1 mm –10 mm residual disease of 16 months (p=0.041) and the historical PFS of 14 months in patients with > 10 mm residual disease. In this study, the median PFS for patients with residual disease < 1cm was 12.9 months and 13.6 months for residual disease > 1 cm. The median PFS and 95% CI of patients after the exclusion of carcinosarcoma and clear cell histology were 13.6 months (8.4, 18.6). Progression-free survival was also determined by MPS score, as demonstrated in **Figure 1B**, but it did not show a statistically significant improvement, with an MPS score > 1 compared to < 1.

All patients underwent germline genetic testing. Pathogenic variants were noted in 3 of the 29 patients: *BARD1*, *BRCA1*, and *BRCA2*.

**Figure 2:**
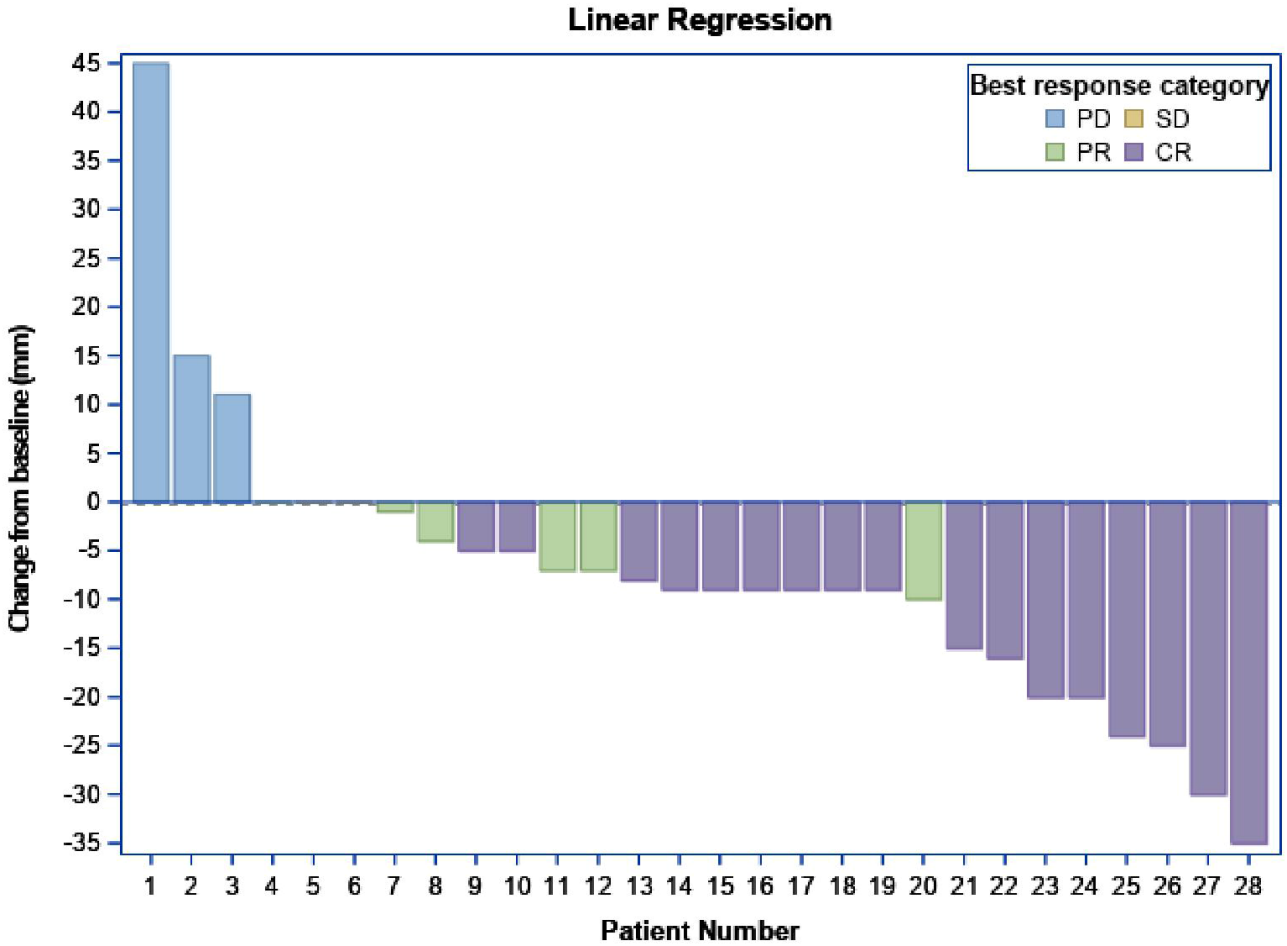
Best response.

The best response to therapy is demonstrated in **Figure 2**. A complete response was noted in 16 patients, stable disease/partial response was noted in 9 patients, and progression of disease was noted in 3 patients.

**Figure 3:**
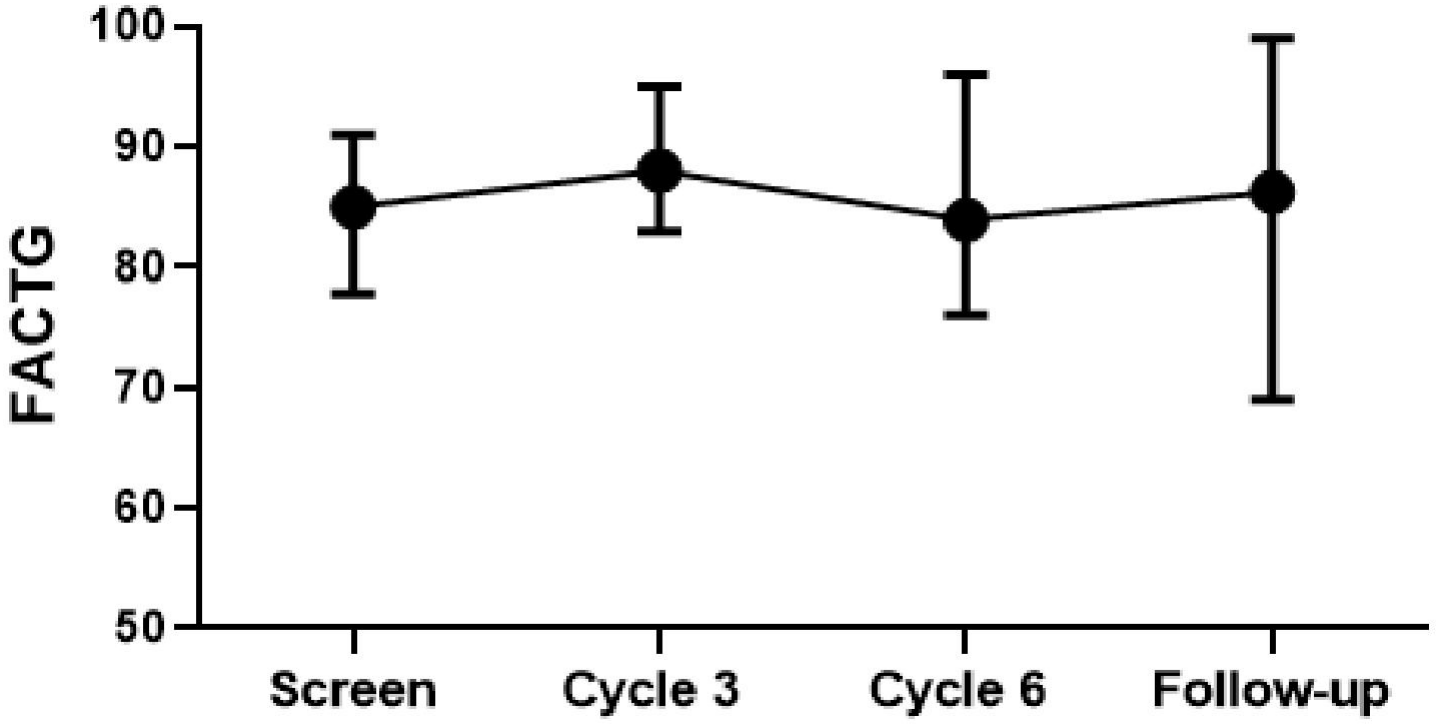
Functional assessment of cancer therapy – general FACT-G scores.

Quality-of-life scores remained high during combination therapy. Compared to the pre-treatment scores, there were no statistically significant changes at 3 months, 6 months, and 18 months after the study (**Figure 3**). However, when compared to the 6-month scores, the 3-month scores were better (p-value=0.041).

## Discussion

### Summary of main results

In our study, we utilized a novel treatment strategy in a poorer prognostic population. Unfortunately, PFS was not significantly impacted by the addition of pembrolizumab to standard platinum–taxane-based therapy followed by pembrolizumab maintenance therapy in patients with incompletely resected, primarily high-grade EOC with predominantly normal germline testing, irrespective of PD-L1 tumor expression.

The literature has a range of PFS for patients with cytoreduced EOC, but in Winter et al.’s (3) retrospective analysis of data from 1895 stage III EOC patients with platinum and paclitaxel combination therapy on six GOG trials, the median PFS was 17 months. Specifically, for patients with residual disease > 1.0 cm, the PFS was 14 months. Approximately one-third of our study participants had > 1.0 cm of residual disease. The current study included all high-grade histology with the exception of one patient with grade 1 endometrioid histology. The current study also included patients with stage IV disease and clear cell and carcinosarcoma histology. After the exclusion of patients with clear cell or carcinosarcoma histology, the median PFS was noted to be 13.6 months.

PD-L1 expression is the most widely adopted predictor of immune check point inhibition. The status of PD-L1 expression is measured by the proportion of PD-L1 expressing tumor cells and/or immune cells. It would stand to reason that a high PD-L1 expression would correlate with greater tumor response and clinical benefit, but this has not been found consistently (23). Several hypotheses to explain this discrepancy have been considered, such as differing cut-off values and scoring systems in IHC detection of PD-L1 expression, differing IHc antibodies among trials, and that upregulation of PD-L1 could be a consequence of causes other than immunity-dependent upregulation (24). Although elevated PD-L1 expression may be a predictor of response in some solid tumors, because it is not noted consistently in tumors demonstrating responses to therapy, it may be that that PD-L1 staining is not the optimal biomarker for patient selection in ICI therapy. Similarly, in our cohort, we did not see a correlation between PD-L1 tumor expression and response to therapy.

### Results in the context of published literature

Effective combinations encompassing immunotherapies, conventional chemotherapies, and targeted therapies are actively being sought to maximize the benefits of systemic treatment. Recently, two larger combination chemotherapy and immunotherapy trials have been completed. The IMagyn050 trial, a randomized phase III trial, investigated the addition of atezolizumab, an anti-PD-L1 antibody, versus the addition of a placebo to platinum-based chemotherapy and bevacizumab in treatment-naïve stage III-IV EOC (25). Anti-PD-L1 maintenance therapy was not included as part of this study. The PFS and interim OS results did not show any significant benefit with the addition of atezolizumab. This study did include some patients with incompletely resected disease and approximately 25% of patients that were planning to receive neoadjuvant chemotherapy. Patients with low-grade histology were included in this study (~10% of patients) as well. Measurements of residual disease were not included in the baseline characteristics of the trial participants. The JAVELIN 100 trial in EOC investigated the use of avelumab, an anti-PD-L1 antibody in frontline treatment of stage II-IV EOC following cytoreductive surgery or in patients who were planned to receive neoadjuvant chemotherapy. They compared frontline standard chemotherapy plus avelumab maintenance therapy versus combination chemotherapy plus avelumab therapy, followed by avelumab maintenance therapy versus standard platinum-based therapy, followed by observation (26). Similarly, the investigators concluded that the addition of ICI to frontline chemotherapy did not improve PFS. Their study included low-grade histology, and the patient characteristics table noted 35 patients with incomplete resection ≤1 cm and 55 patients with incomplete resection ≥1cm on the combination chemotherapy and avelumab followed by avelumab maintenance arm, a population similar to our study. The PFS in all patients in the chemotherapy and avelumab followed by avelumab maintenance arm was 18.1 months, slightly higher than our study. However, direct comparisons are difficult as the histology for these patients in the equivalent arm is not specifically known. They did not include any data on germline mutational status.

Novel immunotherapy therapeutic strategies for HGSOC are still being explored at a rapid pace. The study of molecular profiling of DNA damage repair genes has determined that interference in efficient DNA damage repair (poly(ADP-ribose polymerase (PARP) inhibition) allows for the accumulation of unrepaired DNA, which promotes immune priming through a range of molecular mechanisms and leads to adaptive upregulation of PD-L1 expression (27). It has also been determined that PARP inhibitors modulate the inflammatory immune microenvironment of tumors, possibly adding much-needed support for the anti-tumor response (28). We are continuing to unravel the immune response to cancer, and thus, there is still great untapped potential for ICI, and it may be that combining ICI with PARP inhibition will prove beneficial (29).

### Strengths and weaknesses

The strengths of our study include that it is prospective and reflects a more defined population of incompletely resected, primarily high-grade, patients with predominantly normal germline status. The obvious limitations of our study include its limited number of participants, which precludes the ability to form broad conclusions based on our findings. Our findings do correlate with the larger JAVELIN trial and have some similarities to their population, thus adding to the data on incompletely resected EOC. In our study, all patients received platinum–taxane-based therapy; the regimens did include both dose-dense and every-3-week paclitaxel–carboplatin combinations, which reflects the variability observed in clinical practice but does not contribute to strict uniformity of treatment. The JAVELIN study also included differing paclitaxel administrations.

Although we were able to obtain germline testing on all of our study participants, homologous recombination deficiency testing was not obtained in all participants, given the timing of the study period, and this may have been useful to help assess response to therapy. An additional weakness of our study is the small size. The findings of this small pilot study are not able to influence practice, but they do add additional information to the literature about this group of patients.

### Implications for practice and future research

Immunotherapy as a treatment strategy for cancer comprises several categories in addition to checkpoint inhibition. Additional categories of immune therapy include oncolytic virus therapy, cancer vaccines, cytokine therapy, and adoptive cell transfer. Enhanced patient selection in clinical trials with immune profiling, stratification of treatment arms by biomarkers, and combining multiple immune therapies are already underway in patients with ovarian cancer (30).

Additionally, new research unraveling the tumor microenvironment, where a myriad of events and crosstalk take place between cancer cells and the host stromal cells, will also be instrumental in revealing therapeutic targets and predicting responses to therapy. The innate immune system is a complex network consisting of natural killer cells, eosinophils, basophils, and phagocytic cells (mast cells, neutrophils, monocytes, macrophages, and dendritic cells). The adaptive immune system, equally complex, consists of lymphocytes, including B cells and T cells. Together, these cell populations comprise the host immune system, which plays an important role in recognizing genomic variations that arise as a result of cancer or disease.

The infiltration of the tumor microenvironment by the host’s adaptive and innate immune system cells as they work to combat disease has the ability to modulate tumor progression and responses to immunotherapy (31). Any ability of immunotherapies to influence these cell populations could positively impact the host’s ability to eradicate disease. Closer study of the tumor microenvironment, including differing tumor microenvironments between completely resected and incompletely resected EOC or before and after neoadjuvant chemotherapy, may also provide additional insights into the host immune response and contribute to the evolving landscape of treatment strategies. Understanding all of these complex cellular adaptations is only beginning (32).

As the tumor microenvironment is better understood, so too will our understanding of the pathways that interconnect cell populations. The *PI3K* pathway is an example of an upcoming therapeutic target. It is frequently upregulated in HGSOC and plays an important role in cell survival, chemoresistance, and the preservation of genomic stability, as well as being implicated in many processes of DNA replication and cell cycle regulation (33). Inhibition of PI3K may lead to genomic instability and mitotic collapse through a decrease of the activity of the spindle assembly checkpoint proteins (Aliyuda F, Moschetta M, Ghose A et al. Current Cancer Drug Targets 2023, 23, 433-446). Inhibitors of the PI3K/AKT/mTOR pathway are currently being studied.

## Data availability statement

The raw data supporting the conclusions of this article will be made available by the authors, without undue reservation.

## Ethics statement

The studies involving humans were approved by Medical College of Wisconsin, Cleveland Clinic. The studies were conducted in accordance with the local legislation and institutional requirements. The participants provided their written informed consent to participate in this study.

## Author contributions

DU: Conceptualization, Data curation, Formal analysis, Funding acquisition, Investigation, Methodology, Project administration, Supervision, Writing – original draft, Writing – review & editing. CM: Formal analysis, Investigation, Project administration, Supervision, Writing – original draft, Writing – review & editing. EB: Investigation, Supervision, Writing – review & editing. EH: Investigation, Supervision, Writing – review & editing. PS: Formal analysis, Methodology, Writing – original draft, Writing – review & editing. LZ: Formal analysis, Methodology, Writing – review & editing. JR: Investigation, Supervision, Writing – review & editing. PR: Investigation, Supervision, Writing – review & editing. HM: Formal analysis, Investigation, Supervision, Writing – original draft, Writing – review & editing. RD: Investigation, Supervision, Writing – review & editing. QC: Data curation, Project administration, Writing – review & editing. WB: Data curation, Formal analysis, Investigation, Supervision, Writing – original draft, Writing – review & editing.
